# Adaptive designs in clinical trials: from scientific advice to marketing authorisation to the European Medicine Agency

**DOI:** 10.1186/s13063-018-3012-x

**Published:** 2018-11-20

**Authors:** Olivier Collignon, Franz Koenig, Armin Koch, Robert James Hemmings, Frank Pétavy, Agnès Saint-Raymond, Marisa Papaluca-Amati, Martin Posch

**Affiliations:** 1 European Medicines Agency, 30 Churchill Place, London, E14 5EU UK; 2 Competence Center for Methodology and Statistics, Luxembourg Institute of Health, 1A-B, rue Thomas Edison, L-1445 Strassen, Luxembourg; 3 Section for Medical Statistics, Center for Medical Statistics, Informatics, and Intelligent Systems, Medical University of Vienna, Spitalgasse 23, 1090 Vienna, Austria; 4 Institut für Biometrie, Medizinische Hochschule Hannover, OE 8410, 30625 Hanover, Germany; 5 Medicines and Healthcare Products Regulatory Agency, 151 Buckingham Palace Road, London, SW1W 9SZ UK

**Keywords:** Clinical trials regulation, Interim analysis, Seamless phase II/III, Sample size re-assessment, Stopping for futility

## Abstract

**Background:**

In recent years, experience on the application of adaptive designs in confirmatory clinical trials has accumulated. Although planning such trials comes at the cost of additional operational complexity, adaptive designs offer the benefit of flexibility to update trial design and objectives as data accrue. In 2007, the European Medicines Agency (EMA) provided guidance on confirmatory clinical trials with adaptive (or flexible) designs. In order to better understand how adaptive trials are implemented in practice and how they may impact medicine approval within the EMA centralised procedure, we followed on 59 medicines for which an adaptive clinical trial had been submitted to the EMA Scientific Advice (SA) and analysed previously in a dedicated EMA survey of scientific advice letters. We scrutinized in particular the submission of the corresponding medicines for a marketing authorisation application (MAA). We also discuss the current regulatory perspective as regards the implementation of adaptive designs in confirmatory clinical trials.

**Methods:**

Using the internal EMA MAA database, the AdisInsight database and related trial registries, we analysed how many of these 59 trials actually started, the completion status, results, the time to trial start, the adaptive elements finally implemented after SA, their possible influence on the success of the trial and corresponding product approval.

**Results:**

Overall 31 trials out of 59 (53%) were retrieved. Thirty of them (97%) have been started and 23 (74%) concluded. Nine of these trials (39% out of 23) demonstrated a significant treatment effect on their primary endpoint and 4 (17% out of 23) supported a marketing authorisation (MA). An additional two trials were stopped using pre-defined criteria for futility, efficiently identifying trials on which further resources should not be spent. Median time to trial start after SA letter was given by EMA was 5 months. In the investigated trial registries, at least 18 trial (58% of 31 retrieved trials) designs were implemented with adaptive elements, which were predominantly dose selection, sample size reassessment (SSR) and stopping for futility (SFF). Among the 11 completed trials including adaptive elements, 6 demonstrated a significant treatment effect on their primary endpoint (55%).

**Conclusions:**

Adaptive designs are now well established in the drug development landscape. If properly pre-planned, adaptations can play a key role in the success of some of these trials, for example to help successfully select the most promising dose regimens for phase II/III trials. Interim analyses can also enable stopping of trials for futility when they do not hold their promises. Type I error rate control, trial integrity and results consistency between the different stages of the analyses are fundamental aspects to be discussed thoroughly. Engaging early dialogue with regulators and implementing the scientific advice received is strongly recommended, since much experience in discussing adaptive designs and assessing their results has been accumulated.

## Introduction

In recent years, increasing experience on the application of adaptive designs in confirmatory clinical trials has accumulated. Their popularity is owed in part to their flexibility to adapt the design based on information arising while the trial progresses but also to their potential to reduce costs and the number of patients enrolled. However, the additional planning and operational complexity, including the potential impact of interim analysis on the trial integrity and on the type I error rate^1^ and other operating characteristics (such as power and impact on estimates [1, 2], have been perceived as drawbacks [3–10]. In order to provide regulatory guidance, the European Medicines Agency (EMA) released the “Reflection paper on methodological issues in confirmatory clinical trials planned with an adaptive design” in 2007 [11] and co-organised two workshops on ”adaptive design in confirmatory trials” with the European Federation of Pharmaceutical Industries and Associations (EFPIA) in 2008 and 2009. The US Food and Drug Administration (FDA) also drafted regulatory documents on the same topic in 2010 for medicines and biologics [12] and has recently published another guideline for medical devices [13].

As confirmatory adaptive designs are now routinely used in clinical trials, in addition to regulatory guidance (which focus mainly on planning aspects), reporting guidance in the form of a Consolidated Standards of Reporting Trials (CONSORT) extension is under development [14].

The EMA “Reflection paper on methodological issues in confirmatory clinical trials planned with an adaptive design” (CHMP/EWP/2459/02) [11] defines a study design as adaptive “if the statistical methodology allows the modification of a design element (for example, sample-size, randomization ratio, number of treatment arms) at an interim analysis with full control of the type I error”.

This is the common theme in all three regulatory guidance documents — for a confirmatory (pivotal) clinical trial strict control of the type I error rate is a regulatory pre-requisite for its acceptance. The EMA reflection paper describes the relevant statistical principles but does not propose specific statistical approaches for clinical trials with adaptive elements. A broad range of statistical methods have been developed allowing design modifications at an interim analysis based on interim data without compromising the type I error rate. The first steps towards flexibility were fully pre-specified adaptation rules, e.g. as in group sequential designs [15] or for blinded sample size reassessment (SSR) (e.g. [16–21]). A milestone for confirmatory adaptive design — almost 30 years ago — was the development of fully adaptive designs, which ensure strict control of type I error rate without fully specifying the adaptation rule [22–24]. The EMA does not require a full pre-specification of the adaptation rule; however, it explicitly requires a pre-specification of the adaptive interim analysis and the statistical methods to control the type I error accordingly. A full specification of the adaptive rule would be required to explore operating characteristics such as power or bias but not necessarily to demonstrate type I error rate control. In the beginning focus was given on sample size adaptations, which are now well established if properly implemented. Further developments included research on multiple hypotheses testing with selection of hypothesis at interim; e.g. see [25, 26]. Initially focus was given on adaptive designs with treatment selection at interim (“adaptive seamless phase II/III designs”) [2, 27–29]. Recently adaptive enrichment designs allowing subgroup selection have attracted more and more interest [30–35], which is also related to the advances made in personalised medicines and targeted therapies. Challenging from a methodological point are still adaptive trials based on time-to-event endpoints; e.g. see [36–38]. For a more detailed review on the development of confirmatory trials with adaptive design over the last 25 years, we refer to [3] and its references.

In parallel to methodological developments, sponsors of clinical trials have regularly sought scientific advice (SA) from the EMA to obtain regulatory feedback on adaptive design proposals. A paper published in 2014 by Elsäßer et al. [39] reports on a review of SA letters issued between 1 January 2007 and 8 May 2012 at the EMA discussing medicines whose development was planned using an adaptive trial. Fifty-nine procedures corresponding mainly to phase II and/or III trials were identified and the objective, design and statistical analysis of the corresponding trials discussed. More information about the general aspects of SA procedures can be found on the EMA website (http://www.ema.europa.eu/ema/index.jsp?curl=pages/regulation/general/general_content_000049.jsp&mid=WC0b01ac05800229b9) and in the original survey of Elsäßer et al. [39].

The aim of our article is to follow up these trials and to investigate whether they have actually been conducted and if they concluded. We also retrieved the marketing authorisation application (MAA) supported by the results of the completed trials. Our motivation was to investigate if these trials were still planned using adaptive elements as initially proposed at the time of SA and to evaluate the impact of the adaptive elements on the trial success. Furthermore, we analysed the time elapsed between SA and trial start. Some regulatory perspectives as regards the implementation of adaptive designs in confirmatory clinical trials are proposed before we finish with a discussion and a conclusion.

## Methods

### Follow-up of the 59 original trials

In the original survey, each of the 59 SA procedures included questions relating to a given active substance whose effect was investigated in a single given adaptive trial. Corresponding confirmatory trials were moreover mostly phase III or phase II/III trials (54/59, 92%) [39].

At the cut-off date of 20 April 2016, we linked each of the corresponding 59 adaptive trials to its medicine name using the EMA internal database in which all the MAAs submitted to the EMA are recorded. All the substances that could not be associated to an MAA in this database were then searched within the Springer AdisInsight database in order to enquire about their current status. This registry is a curated database encompassing numerous sources of trials information such as www.ClinicalTrials.gov, European Union (EU) Clinical Trials Register (https://www.clinicaltrialsregister.eu/), scientific articles and press releases. The medical condition, the phase of the trial and the name of the company were used as search terms. Substances from the original survey were then classified as “match” and “no match” depending on the likelihood of associating them to a trial published on the website. Trials corresponding to the substances that could be retrieved were then classified as “completed”, “active, no longer recruiting”, “recruiting”, “not yet recruiting” or “discontinued” according to the AdisInsight glossary. Trials classified as “completed” (i.e. all patients have been enrolled and no more visits are planned) and “discontinued” were categorised as “concluded”. As in the original survey [39], some details about the initial SA procedure (e.g. type of product, therapeutic area), the proposed adaptive study (e.g. type of primary endpoint, phase II or phase III study, initial number of study arms) and the categorisation of Committee for Medicinal Products for Human Use (CHMP)/Scientific Advice Working Party (SAWP) response were collected, differentiating the retrieved trials from those which were not. The start and completion years of the completed trials were also reported.

### Use of adaptive designs

In this paper, we focused only on the analysis of treatment efficacy on the primary endpoint.

For all retrieved trials, available elements indicating the planning of adaptive techniques in the corresponding webpage of AdisInsight or related databases were collected. Substances for which SA about adaptive elements had been requested from the EMA after the trial start were systematically categorised as including adaptations even if not specified in AdisInsight or related databases.

When firstly described during the SA procedure [39], the 59 trials had been classified by the authors of the original survey in order to distinguish trials for which no concerns were raised by CHMP, trials that required further investigation from the sponsor (i.e. “conditionally approved” trials) and trials for which the use of adaptive elements was considered inappropriate by the CHMP. This information was used to cross-tabulate the intended use of adaptive elements in the retrieved trials by their previous acceptance at SA. Finally, for all studies classified as “completed” or “active, no longer recruiting”, the planned and actual sample sizes at the end of the trial were collected in order to discuss them by the planning of sample size re-assessment (SSR) after SA, as reported in AdisInsight.

### Analysis of time lag from SA to trial start

The time from SA letter to trial start was calculated in a time-to-event analysis for all retrieved trials. Following the ClinicalTrials.gov glossary, the trial start date, defined as “the actual date on which the first participant was enrolled in the clinical study” was collected in AdisInsight or related databases for all trials classified as “completed”, “active, no longer recruiting”, “recruiting” or “discontinued”. Trials classified as “not yet recruiting” were considered as censored using the cut-off date of 20 April 2016 as the date of last follow-up. Products whose trial was initiated before SA were imputed 0 month of time to event. Finally, the corresponding Kaplan-Meier curve was plotted.

### Primary endpoint

We investigated whether completed trials (i.e. those in which the last visit of the last patient enrolled has occurred) met their primary endpoint, i.e. demonstrated a significant treatment effect on their primary endpoint (*p* value reported in AdisInsight or related trial registries inferior to 0.05).

### Missing information

Some information, e.g. start date or use of adaptive elements, was sometimes missing in AdisInsight. Information was also searched in ClinicalTrials.gov and in the EU Clinical Trials Register using the corresponding trial identifiers given in AdisInsight.

## Results

Numerous descriptive statistics about the sample of 59 products discussed during SA can be found in the original survey of Elsäßer et al. [39] and are therefore not reported again in this paper. Of note, all the trials were sponsored by pharmaceutical companies.

### Matching of the 59 active substances

**Fig. 1:**
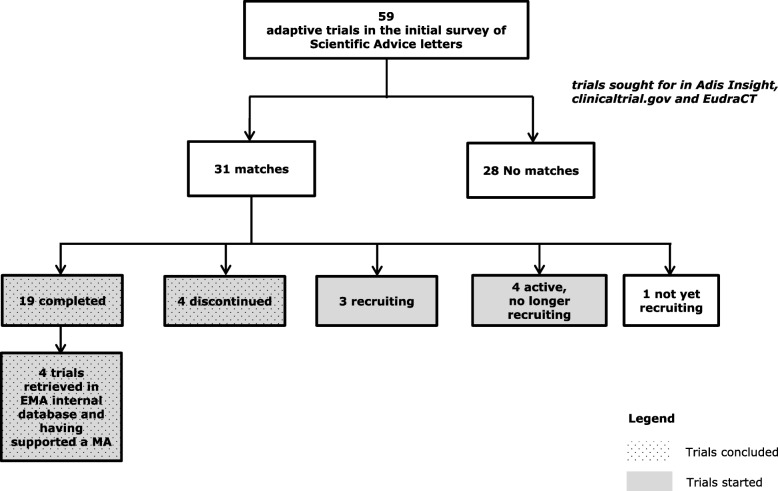
SA letters matching process

The detailed results of the SA trials matching process are given in Fig. 1. A total of 31 trials (31/59, 53%) were retrieved, among which 30 trials (30/31, 97%) have started recruitment, 23 (23/31, 74%) concluded and 4 (4/31, 13%) supported an MA granted by the CHMP. Four of the 23 concluded trials (17%) had been discontinued (2 were stopped according to a pre-planned stopping-for-futility (SFF) analysis and 2 were halted due to difficulty in recruitment).

As in the original survey [39], Table 1 reports numerous descriptive statistics by differentiating the 31 retrieved trials from the 28 trials (28/59, 47%) which were not. No relevant difference which could explain why some of the trials were not retrieved was observed. No other information about the remaining 28 trials and the corresponding active substances was found in the different trials registries. Tables 2 and 3 report respectively the start and completion years of the 30 started trials and the 19 completed ones.

**Table 1:** Descriptive statistics of the 59 scientific advice (SA)/protocol assistance (PA) procedures in the years 2007–2012 from the initial survey [39] categorised by matching status

Variable		31 retrieved trials (*n* (%))	28 non-retrieved trials (*n* (%))
Type of medicinal product	New chemical entity	10 (32%)	13 (46%)
	Known chemical entity	10 (32%)	3 (11%)
	New biological	9 (29%)	4 (14%)
	Known biological	1 (3%)	5 (18%)
	Advanced therapy	1 (3%)	3 (11%)
Therapeutic area of the indication of the medicinal product	Infectious disorders	3 (10%)	1 (4%)
	Oncology	14 (45%)	13 (46%)
	Endocrine and metabolic disorders	1 (3%)	2 (7%)
	Neurologic and psychiatric disorders	2 (6%)	1 (4%)
	Cardiovascular	5 (16%)	5 (18%)
	Diagnostics	2 (6%)	4 (14%)
	Respiratory	0 (0%)	1 (4%)
	Dermatology	2 (6%)	1 (4%)
	Others	2 (6%)	0 (0%)
Rare disease (prevalence of < 5/10,000)		21 (68%)	14 (50%)
Applied for orphan designation		10 (32%)	11 (39%)
Small or medium enterprise		8 (26%)	7 (25%)
Year when the SA/PA letter was issued	2007	4 (13%)	3 (11%)
	2008	7 (23%)	2 (7%)
	2009	3 (10%)	4 (14%)
	2010	7 (23%)	4 (14%)
	2011	8 (26%)	10 (36%)
	2012	2 (6%)	5 (18%)
Scale of measurement of the primary endpoint discussed	Time to event	15 (48%)	13 (46%)
	Binary	12 (39%)	8 (29%)
	Continuous	4 (13%)	7 (25%)
Adaptive study is the only pivotal trial		24 (77%)	20 (71%)
Development phase for which the adaptive clinical trial is proposed	Phase II or IIb	3 (10%)	1 (4%)
	Phase II/III	8 (26%)	8 (29%)
	Phase III	19 (61%)	19 (68%)
	Pediatric study	1 (3%)	0 (0%)
Number of arms of the adaptive trial discussed	1	1 (3%)	1 (4%)
	2	15 (48%)	19 (68%)
	3	9 (29%)	6 (21%)
	> 3	6 (19%)	2 (7%)
Stopping for futility was planned for in the adaptive trial	Yes	17 (55%)	14 (50%)
Stopping for efficacy was planned for in the adaptive trial	Yes	8 (26%)	11 (39%)
Number of interim analyses planned in the adaptive trial	1	21 (68%)	22 (79%)
	2	8 (26%)	5 (18%)
	> 2	2 (6%)	1 (4%)
Type of adaptations planned (multiple answers possible)	Sample size reassessment	19 (61%)	24 (86%)
	Population enrichment	1 (3%)	4 (14%)
	Dropping of treatment arms	13 (42%)	6 (21%)
	Other adaptations	3 (10%)	1 (4%)
CHMP raised issues regarding type I error rate control		14 (45%)	5 (18%)
Categorisation of the CHMP advice regarding the adaptive study design	Accepted	7 (23%)	8 (29%)
	Accepted conditionally (concerns to be addressed)	17 (55%)	15 (54%)
	Not accepted	7 (23%)	5 (18%)

**Table 2:** Start year of the 30 started trials

Start year (*N* = 30)	Number *n* (%)
2006	1 (3%)
2007	4 (13%)
2008	6 (20%)
2009	2 (7%)
2010	6 (20%)
2011	6 (20%)
2012	4 (13%)
2013	0 (0%)
2014	1 (3%)

**Table 3:** Completion year of the 19 completed trials

Completion year (*N* = 19)	Number *n* (%)
2008	1 (5%)
2011	4 (21%)
2012	3 (16%)
2013	6 (32%)
2014	3 (16%)
2015	2 (11%)

### Use of adaptive design

Using the different trial registries, 18 trials (58% of the 31 retrieved trials) were still described as planned with adaptive elements after SA. Of note, 6 of these trials were systematically categorised as including adaptive elements since corresponding SA was requested after study start. The information could not be retrieved for the other 13 remaining products. In Table 4, trials are cross-tabulated by current specified use of adaptive elements and prior acceptance of the planned adaptations at the time of SA letter. Among the retrieved trials, 6/7 (86%) trials for which adaptations were endorsed by SA letter were reported as planned with adaptive elements in the conducted trial, and only 12/24 (50%) when the adaptive design was conditionally approved or not approved in an SA letter.

**Table 4:** Use of adaptive elements as reported in trial registries by their acceptance at scientific advice in retrieved trials

Adaptive elements	Accepted	Conditionally accepted	Not accepted	Total
Reported	6	7	5	18
Not reported	1	10	2	13
Total	7	17	7	31

For the retrieved trials the different types of adaptation mentioned in the trial registries were predominantly dose selection (9 out of 31 adaptations reported (29%)), SSR (8/31 (26%)) and SFF (8/31 (26%)). Other types of adaptations (stopping early for efficacy, stopping central review, population enrichment, Bayesian adaptive randomisation, dropping an arm) were less frequently observed (6/31, 19%). Note that one given trial could include several different types of adaptions (e.g. SSR and SFF).

Furthermore, Table 5 shows the type of adaptation planned at the time of SA letter versus the adaptations actually mentioned in AdisInsight or related databases. A given adaptive element was considered as not implemented if only other types of adaptation were reported. Among the retrieved trials, most of the trials including dose selections discussed at SA were still reported as such in the trial registries after SA (6/8, 75%). In contrast, stopping for early efficacy (SFEE) was reported only once in 8 cases (13%). Less standard adaptations, such as changing the primary analysis from a parametric to a non-parametric one or a change of the type of comparator, which were observed during the first SA survey [39], were never implemented (or the corresponding trial was not retrieved).

**Table 5:** Adaptations planned at the time of scientific advice (SA) letter versus the adaptations implemented in the protocol after SA. A given trial can include several and possibly different types of adaptive elements

Adaptations	Planned at the time of SA opinion letter	Implemented	Not implemented	No adaptations mentioned	Not retrieved
Dose selection (phase II/III)	16	6	0	2	8
Sample size reassessment	43	6	4	9	24
Stopping for futility	31	7	2	8	14
Stopping for early efficacy	19	1	2	5	11

**Fig. 2:**
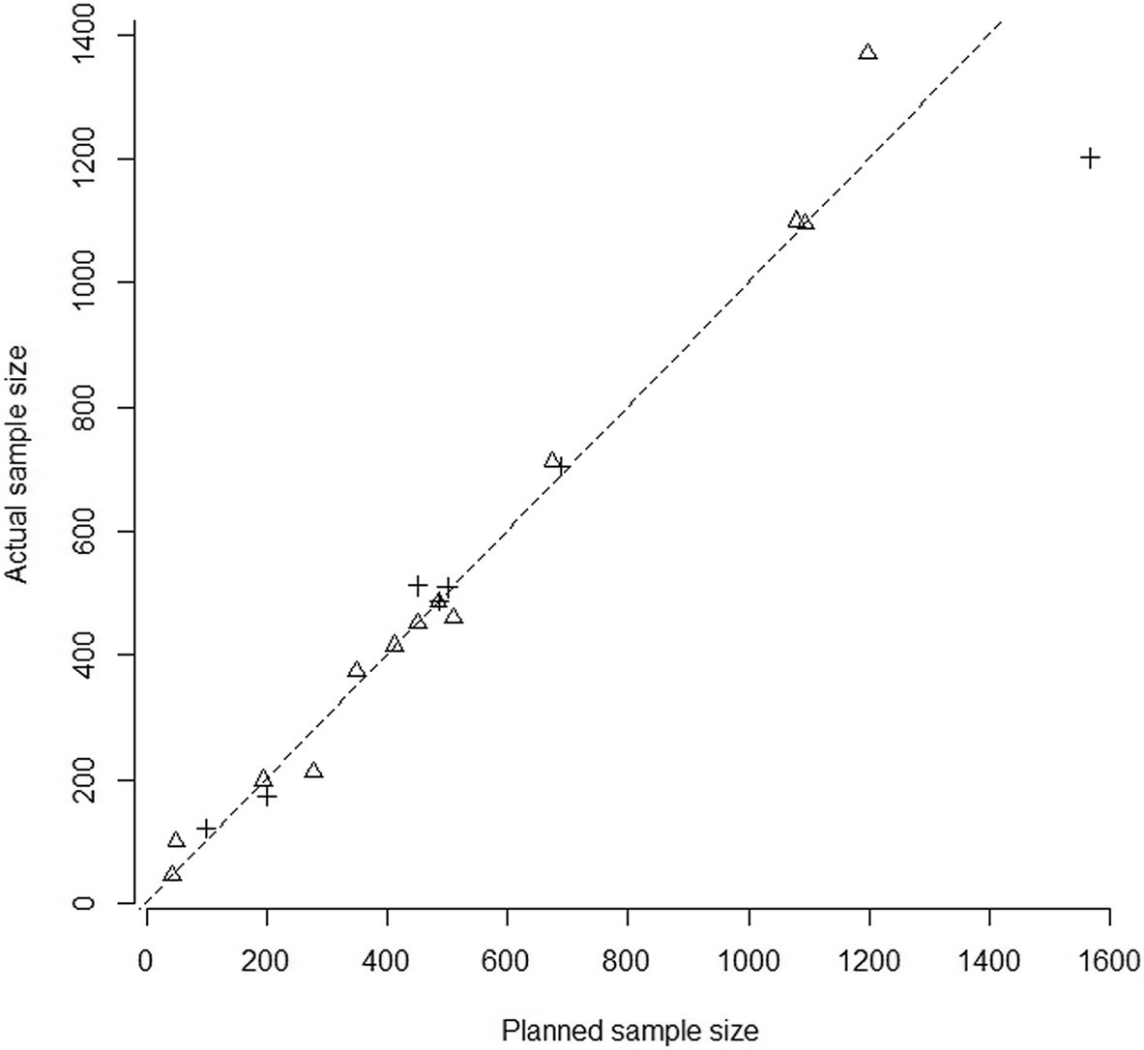
Scatterplot of the planned sample size and of the actual size of retrieved studies classified as “completed” or “active, no longer recruiting” (*n* = 21 with both sample sizes available) as reported in AdisInsight. *Dotted line* represents the line of equality. *Crosses* represent trials planned with SSR after SA, whereas *triangles* represent those which were not

Among the 31 retrieved studies, the average planned sample size was 553 (standard deviation (SD) = 535) (for 3 studies the planned sample size was not reported). For the 23 studies that were approved or classified as “completed” or “active, no longer recruiting”, the scatterplot of the planned sample sizes against the actual ones is given in Fig. 2 (21 studies with both sample sizes reported) and categorised by actual planning of SSR after SA. This shows that planned and actual sample sizes were almost always very close to each other, even when an SSR was initially envisaged. Interestingly, one of the trials planned with SSR allowed reduction of the sample size from 1566 to 1202.

### Analysis of time from SA to trial start

**Fig. 3:**
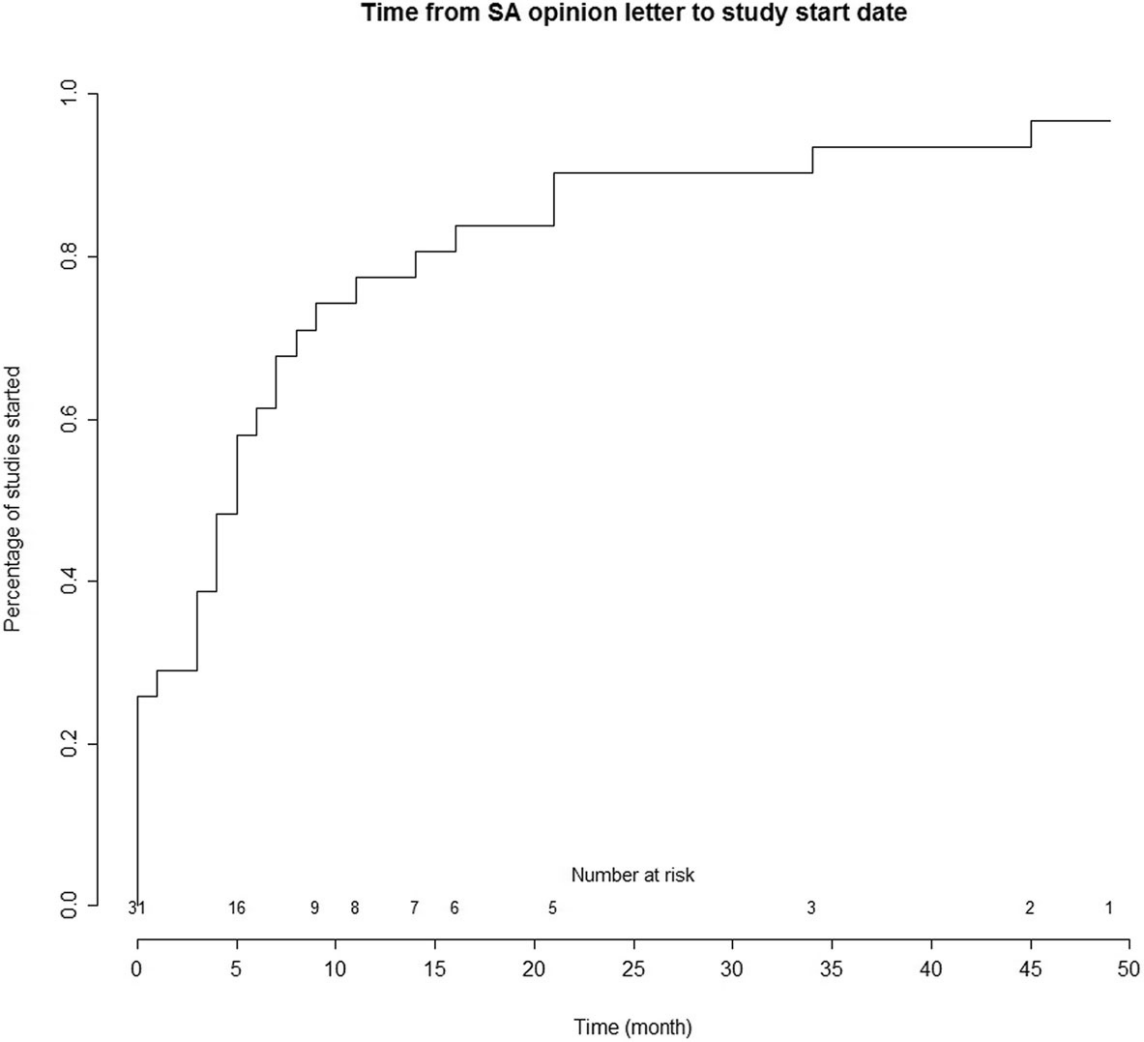
Kaplan-Meier curve of the time from SA letter to trial start date (*n* = 31)

Time-to-event analysis showed that more than 50% of the 31 retrieved trials started within 5 months after the SA letter was given (Fig. 3). Of note, 6/59 SA procedures requested after the start of the trial were imputed zero month of time to event.

### Primary endpoints

Among the 19 completed trials, 9 (47%) met their primary endpoint while the 10 (53%) others did not or no information about the significance of the treatment effect on their primary endpoint was reported. Among completed trials planned with adaptive elements, 6 out of 11 met their primary endpoint (55%).

Two trials were discontinued according to pre-planned SFF analysis respectively about 16 and 51 months after start of enrolment.

### Impact of the adaptations on trial outcome

Four trials supported an MA granted by the CHMP in the context of the centralised procedure.^2^ Two trials included especially interesting adaptations.

The first trial design was a multicentre, double-blind, randomised controlled adaptive phase II/III clinical trial with four groups of experimental treatment regimens and a placebo arm in proliferating haemangiomas in infants. The primary endpoint of the trial was the percentage of complete or nearly complete resolution of the haemangioma. An interim analysis was planned (stage 1) in order to allow an Independent Data Monitoring Committee (IDMC) to discontinue the enrolment in the less promising treatment regimen(s), to re-assess the sample size (based on conditional power) or to stop the trial for futility. The type I error rate of the overall procedure was controlled at 0.005 (using the closed-test principle, the Simes method and a weighted inverse normal combination function [40]). This small alpha level was suggested in an early discussion with the US FDA in order to provide stronger evidence of the treatment effect since the MA was based on a single pivotal trial (see summary of the discussion with the US FDA in [41]).

In total 460 patients were randomised. On the intention-to-treat (ITT) sample interim analysis was conducted after the first 188 were followed up. The highest dose, administered for the longest period of time (regimen 4), showed a significantly higher percentage of haemangioma resolution compared to placebo (63% versus 8%, *p* < 0.001) and was the only selected regimen for the second stage. The sample size was not increased. After the second stage, the respective percentages of response were 60% and 4% for the selected experimental treatment regimen and placebo (*p* < 0.001, adjusted for multiplicity, including all patients randomised in the first stage and the patients randomised to the selected treatment regimen and placebo in the second stage). More results of this trial as well the statistical analysis plan can be found in [40, 42] and their online supplementary material. An extensive discussion about the trial design and logistic issues has been published recently as a book chapter [41]. In particular, it includes an interesting report on the discussions with regulators. The European Public Assessment Report (EPAR) can be found online [43].

The second trial was an adaptive, seamless, phase II/III, multicentre, randomised, placebo-controlled, double-blind clinical trial comparing seven different doses of dulaglutide to sitagliptin and to placebo in patients with diabetes mellitus of type II (T2DM) treated with metformin. The primary objective was to choose up to two safer and more efficacious doses and demonstrate the non-inferiority of the high dose of dulaglutide to sitagliptin. The primary endpoint was glycemic control measured by change from baseline measurement of haemoglobin A_1c_ (HbA_1c_).

This trial was separated into two stages encompassing several adaptive elements. During the first stage, the randomisation probabilities were updated every 2 weeks as data accrued. When enough information was available, only the two best doses would be pursued to stage 2 with the placebo (using a fixed randomisation ratio). The trial would have to be stopped for futility if no safe and efficacious dose could be chosen. The sample sizes of the first stage and of the final analysis were not fixed and could also be adapted.

All these adaptations were performed using a complex Bayesian algorithm relying on a composite measure of efficacy and safety called the clinical utility index (CUI), which comprises functions modelling the effect of the medicine on HbA_1c_, weight, diastolic blood pressure and heart rate as compared to placebo.

An independent statistical analysis centre (SAC) and IDMC were responsible for the interim analyses. A tree gatekeeping procedure was used to control the type I error rate; however, the extensive adaptive nature of this trial required further investigations [44].

At the end of the first stage (dose finding and adaptive randomisation), two dose regimens were selected. At the end of the second stage in the ITT population, both dulaglutide doses were non-inferior to sitagliptin (least square (LS) mean difference − 0.71% [− 0.87; − 0.55] and − 0.47% [− 0.63; − 0.31] respectively, *p* < 0.001, adjusted for multiplicity). Both dulaglutide doses were also significantly superior to sitagliptin (*p* < 0.001, adjusted for multiplicity). In order to support the MAA, the results of four subsequent phase III trials (whose investigated doses were elicited by this adaptive phase II/III trial) were concurrently submitted and exhibited a consistent and significant effect on HbA_1c_ change from baseline as compared to placebo and active comparator. The design and results of this trial have been described extensively in the literature [45–48]. Its EPAR is available online [49].

In these two phase II/III trials, the dose selection stage was essential to the success of the MAA. In the second example this especially enabled one to choose the dose to be further re-investigated in the other jointly submitted phase III trials. Information on the two other trials having supported an MA can be found in the literature [50, 51] as well as on the EMA website [52–54].

Adaptive trials can also be useful to avoid continuing the development of ineffective drugs. For example, in the four discontinued studies from Fig. 1, two were stopped according to a pre-planned SFF analysis respectively about 16 and 51 months after start of enrolment (the two others were halted due to difficulty in recruitment). The first phase II trial in type III epidermal growth factor receptor mutation (EGFRvIII)-expressing glioblastoma used overall survival as an endpoint. The second trial was a phase III trial in patients with metastatic or locally advanced and unresectable chondrosarcoma with progression-free survival as an endpoint. This information was extracted from AdisInsight, which indicated that the SFF analysis was pre-planned for both trials.

## Regulatory aspects

In this section we discuss the most frequent regulatory comments on the use of adaptive elements raised by the CHMP during SA or the assessment of the centralised procedure, as illustrated with the four medicines having reached MAA. Some items are not specific to adaptive designs but can apply to standard group sequential designs as well. In the context of drug regulation, the wording “applicants” is often used in this section, referring, e.g. to the body submitting an MAA or requesting an SA procedure to the EMA. This word could easily by substituted by “researcher” or “trialist”, referring to the person designing the protocol and the methodology of a clinical trial.

### Rationale for adaptive designs

The rationale for designing adaptive trials and the objectives of the adaptations should be made explicit by the sponsors, as in principle the acknowledging of limited knowledge contradicts the confirmatory nature of the trials in the late stage of drug development. For example, often for phase III a broader patient population is recruited than in initial proof-of-concept and phase II clinical trials, and it may be uncertain whether promising findings of earlier trials can be replicated in the confirmatory studies to the full extent. Sample size re-calculation is then an option to make use of the accumulating information in the ongoing trial to compensate increases in variability or decreases in the treatment effect.

Although adaptive designs can be beneficial to a trial success, the use of such designs bears risks. In phase II/III trials for example, a loss of “thinking” time between the two stages of the trials can sometimes be seen as a hurdle, and funding both stages upfront can also be problematic. Applicants should be able to weigh these risks as compared to those of a standard development program. In an SA discussion, a sponsor seemed to have underestimated the risks of running a seamless phase II/III trial. The sponsor was thus requested to explain the rationale for planning an adaptive design versus a standard program of separate trials. The justification was that this would allow investigating more doses. In addition, the trial could be stopped in case of serious safety concerns more quickly since safety data would be monitored regularly across both stages of the trial. The seamless nature of the trial would also enable one to report more information on patients randomised during the first stage in a dose group to be continued until the end of the trial with a longer duration of treatment. These considerations — which could of course also be designed into separate trials though by convention are not — have to be balanced against:

- A potential loss in “thinking time” between stages
- The principal option to include the outcome of a phase II trial regarding safety in the assessment of findings in the phase III trial
- Some general considerations about trial efficiency once a larger number of doses is included in the phase II part of the trial and the overall decision-making strategy has to include the outcome of the phase II part of the trial.

It is seen as a strength of the adaptive approach that all these considerations can be investigated and discussed at the planning stage. Obviously, introducing adaptive elements should rely on thorough knowledge of the test medicine and the comparator and should include careful analysis of the target population, the doses and the duration of treatment.

### Type I error rate control

Identifying potential false positive findings is a key element of the assessment of clinical trials results [55]. The risk is higher when adaptive elements are introduced in the design. Specific methods have to be planned in the protocol to avoid the inflation of the pre-specified type I error rate due to the inspection of preliminary results from an interim analysis. For each of the four trials having supported an MA, type I error rate control was discussed at the time of SA. Statistical methods that analytically control the type I error rate are clearly preferred. Adaptive designs for which control of the type I error rate cannot be shown analytically are more controversially discussed [56], such as Bayesian adaptive randomisation [57–59]. A special need, justified from the context of the trial, would be required to justify a design where type I error rate control can only be ensured by means of statistical simulations, in particular where a design would be available in which type I error rate control can be ensured analytically. These types of adaptive designs were rarely implemented as pivotal trials, but interestingly one of the two case studies described later in the manuscript is an example where the first stage of a pivotal trial was implemented with response adaptive elements.

### Trial integrity and consistency of results before and after the interim analysis

Unless treatment assignment can be fully blinded, maintaining the confidentiality of interim results is fundamental to trial integrity, in particular where an adaptation is planned. This avoids having too much knowledge about the average outcome of treatment influences, for example, the recruitment or the assessment of the patients recruited to the second stage (knowing that the experimental treatment is superior to the control may influence the estimation of a physician of a patient’s state if he can guess the treatment allocation of this patient).

The extent of the sponsor’s involvement in decision making remains controversial and is best avoided, so that it is evident that there is no intentional or unintentional communication of results to the treating physicians. Of course, this would be of particular concern where recruitment and results before and after the interim analysis are heterogeneous. Applicants should be able to substantiate that no modifications were made to the trial other than the planned adaptations, and in particular that no changes in patient recruitment, management, assessment or other aspects of trial conduct arose based on the sponsor or the investigator becoming aware of the emerging trial data. A remaining difficulty is when some sponsor personnel are permitted to be aware of interim results but are said to be firewalled from the rest of their organisation. The integrity of the firewall is difficult to substantiate, and if sponsors are to become aware of interim trial data, further research leading to identification of best practice is needed.

In assessments, the CHMP emphasised data integrity as essential to adaptive designs and stated that discordant results between findings obtained at interim analysis (stage 1) and at completion (stage 2) would raise concerns. Although random fluctuations can generate discrepancies between both stages of the analysis, if the differences are large, the credibility of the trial can be threatened as soon as the trial is not completely blind and dissemination of information may not be controlled. For example, the CHMP reminded some sponsors of the need to present two separate analyses corresponding to the participants included in each stage of the trial, to discuss any observed discrepancies and whether results of both stages could be combined, especially as regards demographics of participants or other aspects that may influence the treatment effect and thereafter complicate the decision.

### Methods

The adaptive elements to be used in the clinical trial should be planned in advance, and the statistical methodology should be fully detailed in the protocol. Although in principle the responsibility for the methodological correctness of a statistical design rests with the applicant, some applicants have been requested to provide more detailed information on statistical methods used with the adaptive design in situations where aspects of type I error rate control or the ability to derive an unbiased estimate of the treatment effect were not obvious to the assessor. In a phase II/III design for example, a sponsor was requested to investigate all the different scenarios of dose selection at interim analysis to better understand the advantages and potential disadvantages of the proposed design. The number and complexity of adaptive elements in a protocol should be limited, since, as explained above, trials in late stage drug development are supposed to confirm findings and knowledge established in earlier development phases.

### Single versus multiple pivotal trials

According to the reflection paper [11], a single phase II/III trial is generally not considered sufficient for an application with only one pivotal trial. Obviously a sound basis for decision making from phase II is a key argument for an application with one pivotal trial. In consequence, the reflection paper recommends that a phase II/III confirmatory clinical trial should be supported by another independent pivotal III trial.

For rare diseases however, a single phase II/III trial is an option, provided it is at least equally optimal from the perspective of sample size requirements. In the case of the treatment of infantile haemangiomas which relied on a single phase II/III trial, the treatment effect was compelling, and the sponsor engaged in early dialogue with regulators through scientific advice and integrated comments into an updated trial protocol. On the other hand, more complex methodologies can be used when the results of other non-adaptive trials are also provided in the application. In one example, the CHMP initially discussed a standard phase II/III trial to define the dose followed by several confirmatory phase III trials and deemed a seamless design unnecessary. The sponsor argued that this seamless trial should be considered in the context of the submission, since it would be accompanied by several non-adaptive confirmatory phase III trials. The CHMP could agree to this argument because even if the integrity of the phase II/III trial were drawn into consideration, sufficient independent support for the chosen dose and other aspects of the treatment modalities would be available for decision making at the approval stage if the benefit was consistently positive across trials.

### Stopping early for efficacy

Stopping a trial for overwhelming efficacy may not be adequate to support MA, even when the result of the primary efficacy endpoint is compelling. Data on safety, on other endpoints or in relevant subgroups of the patient population might not be available at the time of the interim analysis to allow for a proper decision on the benefit/risk for the experimental drug. For one of our four examples, early stopping at interim analysis was not endorsed, because the target population was not homogeneous and sufficient information on internal consistency and benefit/risk in important subgroups would not be generated. Admittedly, there is no difference in this aspect between a trial with a standard interim analysis and a trial planned and conducted with an adaptive design. Sponsors should always bear in mind that medicines are usually only licensed after a thorough assessment of the overall evidence of effectiveness and not based on an isolated significant finding.

### Stopping for futility

Trials can sometimes be stopped for futility because of a disappointing outcome regarding efficacy and/or safety. This is important to avoid unnecessary exposure of additional patients to an experiment. This is an important consideration in any clinical trial that should be encouraged, but it should be balanced against the premature nature of interim data that may mislead decision making. Importantly, as stated above, an assessment of benefit and risk is needed before stopping a trial, because in some instances smaller treatment effects may be well justified if safety is particularly compelling for a new drug.

## Discussion

In our sample more than half (58%) of the retrieved trials were reported in the different trial registries as planned with adaptive elements after the SA letter was given. Since the planning of adaptive elements is not systematically recorded in the AdisInsight database or in the related trial registries, this figure must thus be considered as a lower bound. Trial registries, indeed, fail to offer convenient reporting options of the trial design elements, which makes it difficult to disentangle trials for which adaptations were modified or abandoned after the SA letter from underreporting. Dose selection, SSR and SFF are now part of the regulatory landscape and were planned with variable levels of complexity in this survey. On the contrary, less standard adaptations such as changing the primary analysis, population enrichment and Bayesian adaptive randomisation are still less often encountered. Of note, the latter is generally not encouraged, because if by random chance the first patients enrolled in the placebo arm have a high response, most of the subsequent patients would also be likely to be randomised in the placebo arm. In the second case study discussed in this paper, the controversial use of this methodology was mitigated by the fact that there were several other phase III trials submitted in the package.

The findings elicited in this article have to be tempered by the still limited number of trials, under-reporting and missing information about trial design in the public trial databases. Overall it was a challenging exercise to track down the 59 original adaptive trials discussed by the EMA Scientific Advice Working Party (SAWP) since sponsors are not obliged to notify EMA when a trial starts or to report its outcome if the related medicine has not been submitted for approval. Moreover, for retrieved trials the statistical significance of the treatment effect on the primary endpoint was not always reported in AdisInsight or related trial registries. Also, when the corresponding *p* value was reported, it was in general unclear if it was accounting for the multiplicity due to the adaptations. In addition, reporting on the intended use of adaptive elements is not mandated by public databases, and there is no field available for sponsors to record adaptive elements of the design. In a survey of the adaptive designs registered in ClinicalTrials.gov, other authors recently highlighted the inappropriateness of this database to report adaptive characteristics included in some trials (e.g. SSR, stopping rules) and thus to retrieve the adaptive designs [60]. In the 31 retrieved trials the median time to trial start after SA was 5 months. In order to put this figure in context and compare it to trials which were planned without adaptations, it would be interesting to plan a prospective dedicated case-control study based on SA letter submissions aiming at comparing time to trial start between adaptive designs and non-adaptive ones. Of note, this analysis would have to be adjusted for confounders such as therapeutic area for example.

The main regulatory checkpoints for adaptive designs include the justification for choosing complex adaptive rather than standard designs, the control of the type I error rate, data integrity and results consistency across stages of the adaptive design. Others, including a recent retrospective survey of adaptive designs from the US FDA, encouraged similar practices [4, 61]: the rationale of planning an adaptive trial rather than a simpler design, thorough explanation of the adaptations planned (with detailed statistical analysis plan), control of the type I error rate and minimisation of operational bias to maintain data integrity during interim analysis.

## Conclusions

Although only a limited number of trials could be retrieved for this analysis, we confirm that properly pre-planned adaptive designs are not a barrier to regulatory approval. Provided that the adaptive elements are properly planned, justified, conducted and reported with diligence, adaptive trials can be considered by sponsors to support the demonstration of benefit/risk balance [60]. Type I error rate control, trial integrity and results consistency should especially be discussed thoroughly, and it is particularly important to provide reassurance that the integrity of the trial has not been damaged. Adaptive designs can be beneficial to the trial success and interim analyses. They can also enable sponsors to stop a disappointing trial for toxicity or futility, but it is definitely clear that a commitment to higher investments at the planning stage is needed. As also recommended by the US FDA [61], early dialogue with regulators through SA is necessary so that potential issues can be solved before the start of the trial, and in order to facilitate approval later.

## Abbreviations

AD: Adaptive design
CHMP: Committee for Medicinal Products for Human Use
CUI: Clinical utility index
EFPIA: European Federation of Pharmaceutical Industries and Associations
EGFRvIII: Type III epidermal growth factor receptor
EMA: European Medicines Agency
EPAR: European Public Assessment Report
FDA: Food and Drug Administration (Agency)
HbA_1c_: Haemoglobin A_1c_
IDMC: Independent Data Monitoring Committee
ITT: Intention to treat
LS: Least square
MA: Marketing authorisation
MAA: Marketing authorisation application
SA: Scientific advice
SAC: Statistical analysis centre
SAWP: Scientific Advice Working Party
SD: Standard deviation
SFEE: Stopping for early efficacy
SFF: Stopping for futility
SSR: Sample size re-assessment
T2DM: Diabetes mellitus of type II

## Footnotes

1 As mentioned in the European Medicines Agency guideline on multiplicity issues in clinical trials EMA/CHMP/44762/2017, “the term ‘control of type I error’ rate will be used as an abbreviation for the control of the study-wise type I error in the strong sense, i.e. there is control on the probability to reject at least one out of several true null hypotheses, regardless of which subset of null hypotheses happens to be true.”

2 Under the centralised authorisation procedure, pharmaceutical companies submit a single MA to the EMA in order to market the medicine in all EU member states as well as in the European Economic Area (EEA) countries Iceland, Liechtenstein and Norway.
